# Synthesis of Chalcones with Anticancer Activities

**DOI:** 10.3390/molecules17066179

**Published:** 2012-05-25

**Authors:** Suvitha Syam, Siddig Ibrahim Abdelwahab, Mohammed Ali Al-Mamary, Syam Mohan

**Affiliations:** 1 UPM-MAKNA Cancer Research Lab, Institute of Bioscience, University Putra Malaysia, 43400 Serdang, Selangor, Malaysia; Email: suvithaavs@gmail.com; 2 Department of Pharmacy, Faculty of Medicine, University of Malaya, 50603 Kuala Lumpur, Malaysia; Email: siddigroa@yahoo.com; 3 Department of Chemistry, Faculty of Science and Arts in Al-Mukhwah, Al-Baha University, Al-Baha 65431, Saudi Arabia; Email: almamarym@hotmail.com; 4 Centre for Natural Products and Drug Discovery (CENAR), Department of Pharmacology, Faculty of Medicine, University of Malaya, 50603 Kuala Lumpur, Malaysia; Email: syammohanm@yahoo.com

**Keywords:** chalcone, synthetic, cytotoxicity, MCF-7 cells, apoptosis, high content screening, caspase, ROS

## Abstract

Several chalcones were synthesized and their *in vitro* cytotoxicity against various human cell lines, including human breast adenocarcinoma cell line MCF-7, human lung adenocarcinoma cell line A549, human prostate cancer cell line PC3, human adenocarcinoma cell line HT-29 (colorectal cancer) and human normal liver cell line WRL-68 was evaluated. Most of the compounds being active cytotoxic agents, four of them with minimal IC_50_ values were chosen and studied in detail with MCF-7 cells. The compounds **1**, **5**, **23**, and **25** were capable in eliciting apoptosis in MCF-7 cells as shown by multiparameter cytotoxicity assay and caspase-3/7, -8, and -9 activities (*p* < 0.05). The ROS level showed 1.3-fold increase (*p* < 0.05) at the low concentrations used and thus it was concluded that the compounds increased the ROS level eventually leading to apoptosis in MCF-7 cells through intrinsic as well as extrinsic pathways.

## 1. Introduction

The burden of cancer is increasing across the World and thus it is the leading cause of deaths in economically developed countries and second leading cause of deaths in developing countries [1]. Cancer is considered to be one of the most intractable diseases because of the innate characteristics of cancer cells to proliferate uncontrollably, avoid apoptosis, invade and metastasize [2]. Despite the advances in chemotherapy, there are no sufficient clinically useful cytotoxic agents that selectively targets cancer cells.

Most of the chemotherapeutic agent have anticancer activity thanks to their capacity to elicit apoptosis [3]. The physiologically determined cell death, apoptosis, is necessary to maintain tissue homeostasis; where homeostasis refers to the balance between cell proliferation and cellular loss. Since it provides a mechanism of autodigestion for cells that are not functioning properly, the screening of anticancer agents for chemotherapy is designed to identify agents that selectively kill tumor cells. Many natural as well as synthetic agents have demonstrated to elicit apoptosis in cancer cells [4,5,6,7]. Synthetic derivatives are often found to be more active than parent compounds [6,7,8,9].

Chalcones, considered as the precursors of flavonoids and isoflavonoids are widely present in edible plants. Chemically, they consist of open-chain flavonoids in which the two aromatic rings are joined by a three-carbon α,β-unsaturated carbonyl system. Among the flavonoids, chalcones are an interesting target class of compounds which are extensively investigated due to their broad spectrum of biological activities, including anti-inflammatory [10], anti-invasive [11] and antitumour [12] and antibacterial [13] properties. They are regarded as promising anticancer agents against most human cancers. Previous literature suggests that chalcones are capable of inducing apoptosis [14,15] and also have the ability to uncouple mitochondrial respiration and thus collapse mitochondrial membrane potential [16]. Since a number of clinically useful anticancer drugs have genotoxic effects due to their interaction with the amino groups of nucleic acids, chalcones may be devoid of this important side effects [17]. In the ongoing search for potent and selective cytotoxic chalcones, we aimed to synthesize some chalcones and investigate their cytotoxic potential against some cancer cell lines, and also study the detailed mechanism of their cytotoxic activity. The synthesis of chalcones mentioned in this report can be done in simple steps with most of the compounds being produced in >50% overall yield.

## 2. Results and Discussion

### 2.1. Synthesis of Chalcones

**Figure 1:**
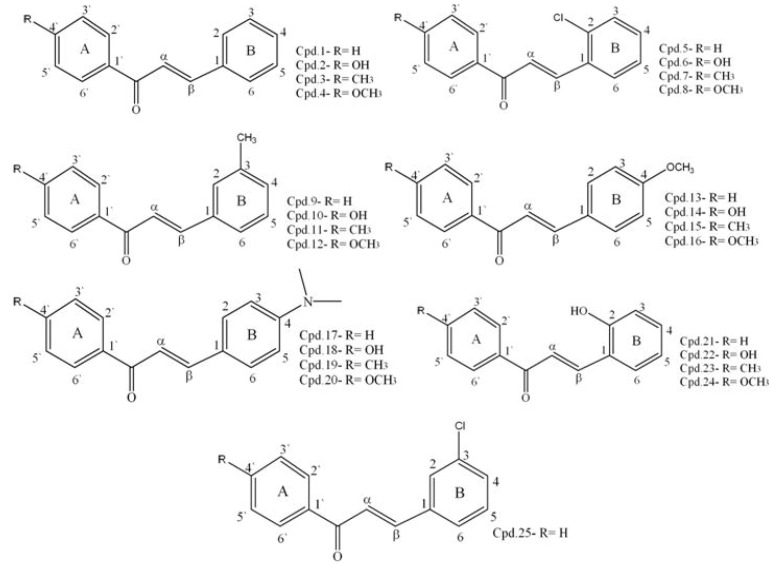
Structures of different chalcones synthesized in this study. ‘Cpd’ stands for an abbreviation of “Compound”.

Different chalcone derivatives were obtained as indicated in Figure 1. The reactants used, chemical namea and formulae of the compounds are detailed in Table 1. The melting points, IR spectra (KBr), cm^−1^ and ^1^H-NMR spectra (δ, ppm) of the compounds were determined (refer to Table 2). Compounds **1**, **5**, **11–13**, **15–17**, **20** and **25** were obtained in high yields ranging between 75–90%; amongst which compound **15** and **20** had the maximum yield, reaching up to 90%. Compounds **3**, **4**, **7–9**, **19** and **21** had yields ranging from 50–74% whilst compounds **2**, **6**, **10**, **14**, **18**, **22–24** showed low yields of less than 50%.

**Table 1:** Chemical data for compounds **1–25**.

No	Reactants	Chalcone (Chemical name)	Formula
1	Acetophenone + Benzaldehyde	1,3-Diphenylpropenone	C_15_H_12_O
2	4H Acetophenone + Benzaldehyde	1-(4-Hydroxyphenyl)-3-phenylpropenone	C_15_H_12_O_2_
3	4-Methylacetophenone + Benzaldehyde	3-Phenyl-1- *p*-tolylpropenone	C_16_H_14_O
4	4-Methoxyacetophenone + Benzaldehyde	1-(4-Methoxyphenyl)-3-phenylpropenone	C_16_H_14_O_2_
5	Acetophenone + 2-Chloro-benzaldehyde	3-(2-Chlorophenyl)-1-phenylpropenone	C_15_H_11_OCl
6	4H Acetophenone + 2-Chloro-benzaldehyde	3-(2-Chlorophenyl)-(4-hydroxyphenyl)-propenone	C_15_H_11_O_2_Cl
7	4-Methylacetophenone + 2-Chloro-benzaldehyde	3-(2-Chlorophenyl)-1-P-tolylpropenone	C_16_H_13_OCl
8	4-Methoxyacetophenone + 2-Chloro-benzaldehyde	3-(2-Chlorophenyl)-1-(4-methoxy-phenyl)propenone	C_16_H_13_O_2_Cl
9	Acetophenone + 3-Methyl-benzaldehyde	1-Phenyl-3- *m*-tolylpropenone	C_16_H_14_O
10	4H Acetophenone + 3-Methyl-benzaldehyde	1-(4-Hydroxyphenyl)-3- *m*-tolylpropenone	C_16_H_14_O_2_
11	4-Methylacetophenone + 3-Methyl-benzaldehyde	3- *m*-Tolyl-1-*p*-tolylpropenone	C_17_H_16_O
12	4-Methoxyacetophenone + 3-Methyl-benzaldehyde	1-(4-Methoxyphenyl)-3- *m*-tolylpropenone	C_17_H_16_O_2_
13	Acetophenone + Anisaldehyde	3-(4-Methoxyphenyl)-1-phenylpropenone	C_16_H_14_O_2_
14	4H Acetophenone + Anisaldehyde	1-(4-Hydroxyphenyl)-3-(4-methoxyphenyl)-propenone	C_16_H_15_O_3_
15	4-Methylacetophenone + Anisaldehyde	3-(4-Methoxyphenyl)-1- *p*-tolylpropenone	C_17_H_16_O_2_
16	4-Methoxyacetophenone + Anisaldehyde	1,3-Bis-(4-Methoxyphenyl)-propenone	C_17_H_16_O_3_
17	Acetophenone + 4-Dimethylamino- benzaldehyde	3-(4-Dimethylaminophenyl)-1-phenyl-propenone	C_17_H_17_NO
18	4H Acetophenone + 4-Dimethylamino-benzaldehyde	3-(4-Dimethylaminophenyl)-1-(4-hydroxy-phenyl)-propenone	C_17_H_17_NO_2_
19	4-Methylacetophenone + 4-Dimethylamino- benzaldehyde	3-(4-Dimethylaminophenyl)-1- *p*-tolyl-propenone	C_18_H_19_NO
20	4-Methoxyacetophenone + 4-Dimethylamino-benzaldehyde	3-(4-Dimethylaminophenyl)-1-(4-methoxy-phenyl)-propenone	C_18_H_19_NO_2_
21	Acetophenone + Salicylaldehyde	3-(2-Hydroxyphenyl)-1-phenylpropenone	C_15_H_12_O_2_
22	4H Acetophenone + Salicylaldehyde	3-(2-Hydroxyphenyl)-1-(4-hydroxyphenyl)-propenone	C_15_H_12_O_3_
23	4-Methylacetophenone + Salicylaldehyde	3-(2-Hydroxyphenyl)-1- *p*-tolylpropenone	C_16_H_14_O_2_
24	4-Methoxyacetophenone + Salicylaldehyde	3-(2-Hydroxyphenyl)-1-(4-methoxyphenyl)-propenone	C_16_H_14_O_3_
25	Acetophenone + 3-Chlorobenzaldehyde	3-(3-Chlorophenyl)-1-phenylpropenone	C_15_H_11_OCl

**Table 2:** Spectral data, yield and melting point of compounds 1–25.

Cpd.	IR-Spectrum (KBr), сm^−1^	^1^H-NMR spectrum (δ, ppm)	Yield (%)	m.p. (°C)
1	3030 (C-H aromatic), 1664 (C=O), 1598 (C=C)	7.77 (d, 1Hα), 8.08 (d, 1Hβ), 7.4–8 (m, 10H, Ar-H)	70–80	56–57
2	3083 (C-H aromatic), 1680 (C=O), 1580 (C=C), 3380 (-OH)	7.56 (d, 1Hα), 7.99 (d, 1Hβ), 7.04–7.9 (m, 9H, Ar-H), 12.9 (s, 1H, -OH)	35–45	120–121
3	3030 (C-H aromatic), 1649 (C=O), 1598 (C=C)	7.38 (d, 1Hα), 8.1 (d, 1Hβ), 7.5–7.9 (m, 9H, Ar-H), 2.29 (s, 3H, -CH_3_)	70–73	70–72
4	3047 (C-H aromatic), 1649 (C=O), 1603 (C=C), 1128 (-OCH_3_)	7.19 (d, 1Hα), 8.2 (d, 1Hβ), 7.29–7.9 (m, 9H, Ar-H), 3.79 (s, 3H, -OCH_3_)	70–74	108–110
5	3047 (C-H aromatic), 1664 (C=O), 1608(C=C), 829 (Ar-Cl)	7.9 (d,1Hα), 8.2 (d, 1Hβ), 7.39–8 (m, 9H, Ar-H)	75–80	53–54
6	3452 (-OH), 1690 (C=O), 1613 (C=C), 829 (Ar-Cl)	7.5 (d, 1Hα), 8.19 (d, 1Hβ), 6.4–8 (m, 8H, Ar-H), 12.7 (s, 1H, -OH)	40–43	191–193
7	3052 (C-H aromatic), 1664 (C=O), 1598 (C=C), 829 (Ar-Cl)	7.9 (d, 1Hα), 8.19 (d, 1Hβ), 7.3–8.1 (m, 8H, Ar-H), 2.3 (s, 3H, -CH_3_)	70–75	50–51
8	3034 (C-H aromatic), 1664 (C=O), 1590 (C=C), 1130 (-OCH_3_), 828 (Ar-Cl)	7 (d, 1Hα), 7.7 (d, 1Hβ), 6.9–8.1 (m, 8H, Ar-H), 3.79 (s, 3H, -OCH_3_)	60–65	124–126
9	3057 (C-H aromatic), 1664 (C=O), 1590 (C=C)	7.19 (d,1Hα), 8.2 (d, 1Hβ), 7.2–8 (m, 9H, Ar-H), 2.3 (s, 3H, -CH_3_)	68–70	68–70
10	3457 (-OH), 3011 (C-H aromatic), 1685 (C=O), 1591 (C=C)	7.2 (d, 1Hα), 7.7 (d, 1Hβ), 7.1–8 (m, 8H, Ar-H), 12.8 (s, 1H, -OH), 2.3 (s, 3H, CH_3_)	30	114–116
11	3027 (C-H aromatic), 1654 (C=O), 1593 (C=C)	7.8 (d, 1Hα), 8.2 (d, 1Hβ), 7.1–8 (m, 8H, Ar-H), 2.3 (s, 3H, -CH_3_)	84	89–91
12	3011 (C-H aromatic), 1659 (C=O), 1588 (C=C), 1126 (-OCH_3_)	7.19 (d, 1Hα), 8.21 (d, 1Hβ), 6.9–8 (m, 8H, Ar-H), 3.8 (s, 3H, -OCH_3_), 2.96 (s, 3H, CH_3_)	80	74–75
13	3057 (C-H aromatic), 1659 (C=O), 1588 (C=C), 1168 (-OCH_3_)	7 (d, 1Hα), 8.2 (d, 1Hβ), 6.9–8.2 (m, 9H, Ar-H), 3.8 (s, 3H, -OCH_3_)	80	77–78
14	3380 (-OH), 3035 (C-H aromatic) 1660 (C=O), 1590 (C=C), 1166 (-OCH_3_)	6.9 (d, 1Hα), 7.9 (d, 1Hβ), 6.7–8.1 (m, 8H, Ar-H), 12.8 (s, 1H, -OH), 3.8 (s, 3H, -OCH_3_)	35	179–181
15	3080 (C-H aromatic), 1649 (C=O), 1588 (C=C), 1170 (-OCH_3_)	7.7 (d, 1Hα), 8.2 (d, 1Hβ), 6.9–8.2 (m, 8H, Ar-H), 2.29 (s, 3H, -CH_3_), 3.8 (s, 3H, -OCH_3_)	85–90	99–100
16	3033 (C-H aromatic), 1659 (C=O), 1588 (C=C), 1168 (-OCH_3_)	7.7 (d, 1Hα), 8.2 (d, 1Hβ), 6.9–8.2 (m, 8H, Ar-H), 3.8(s, 3H, -OCH_3_), 3.8 (s, 3H, OCH_3_)	80–88	102–103
17	3010 (C-H aromatic), 1654(C=O), 1562(C=C), 1340 (C-N)	6.7 (d, 1Hα), 8.21 (d, 1Hβ), 7.3–7.8 (m, 9H, Ar-H), 2.9 (s, 6H, N-(CH_3_)_2_)	80	111–113
18	3380 (-OH), 3012 (C-H aromatic), 1664 (C=O), 1598 (C=C), 1372 (C-N)	6.8 (d, 1Hα), 8.7 (d, 1Hβ), 6.8–8.8 (m, 8H, Ar-H), 9.7 (s, 1H, -OH), 2.99 (s, 6H, N-(CH3)2)	47	76–78
19	3083 (C-H aromatic), 1649 (C=O), 1603 (C=C), 1360 (C-N)	6.7 (d, 1Hα), 8.21 (d, 1Hβ), 7.3–8 (m, 8H, Ar-H), 2.98 (s, 6H, N-(CH_3_)_2_), 2.3 (s, 3H, -CH_3_)	70	124–125
20	3010 (C-H aromatic), 1650 (C=O), 1593 (C=C), 1161 (-OCH3), 1330 (C-N)	6.7 (d, 1Hα), 8.2 (d, 1Hβ), 6.7–8.1 (m, 8H, Ar-H), 2.9 (s, 6H, N-(CH_3_)_2_), 3.79 (s, 3H, -OCH_3_)	86–90	129–131
21	1639 (C=O), 1557 (C=C), 3206 (-OH), 3010 (C-H aromatic)	7.6 (d, 1Hα), 8.2 (d, 1Hβ), 6.7–8.2 (m, 9H, Ar-H), 10.3 (s, 1H, -OH)	50	154–155
22	1669 (C=O), 1590 (C=C), 3467 (-OH), 3005 (C-H aromatic)	7.8 (d, 1Hα), 7.5 (d, 1Hβ), 6.3–8.3 (m, 8H, Ar-H), 10.2 (s, 1H, -OH), 10.2 (s, 1H, -OH)	8–10	287–289
23	1644 (C=O), 1577 (C=C) , 3216 (-OH), 3030 (C-H aromatic)	6.8 (d, 1Hα), 7.8 (d, 1Hβ), 6.7–8.2 (m, 8H, Ar-H), 10.2 (s, 1H, -OH), 2.28 (s, 3H, -CH_3_)	37–40	165–167
24	3252 (-OH), 3000 (C-H aromatic), 1640 (C=O), 1603 (C=C), 1165 (-OCH3)	7.96 (d, 1Hα), 8.19 (d, 1Hβ), 6.7–8.2 (m, 8H, Ar-H), 10.19 (s, 1H, -OH), 3.69 (s, 3H, OCH3)	45	151–153
25	1654 (C=O), 1603 (C=C), 3052 (C-H aromatic), 823 (Ar-Cl)	7.9 (d, 1Hα), 8.2 (d, 1Hβ), 7.2–8 (m, 9H, Ar-H)	80	78–80

### 2.2. Cytotoxic Screening

The cytotoxicity of the synthesized chalcones were studied using the MTT assay in five human cancer cell lines, including MCF-7 (breast), A549 (lung), PC3 (prostate), HT-29 (colorectal) and WRL68 (liver). The results are listed in Table 3. Compounds **2**, **7**, **8**, **14**, **18**, **20**, **22** showed low cytotoxic effects on all the cell lines. Compounds **1**, **5**, **10**, **23**, **24**, **25** showed high cytotoxicity in all cell lines with IC_50_ concentrations lines, except for the A549 cell line. It is already well known that chalcones (both natural and synthetic of less than 20 µg/mL. Compounds **3**, **11**, **12**, **13**, and **21** also showed IC_50 _values of less than 20 µg/mL in all cell origin) are cytotoxic to cancer cells [18,19,20] and this was confirmed by the data of the current study. 

**Table 3:** Cytotoxicity of compounds on different cell lines.

Compound	Cell lines/IC_50_ values (µg/mL)				
	A549	PC3	MCF-7	HT-29	WRL68
1	16.76 ± 1.08	9.108 ± 0.9	6.875 ± 0.219	10.1 ± 1.01	10.55 ± 0.89
2	>100	>100	>100	>100	>100
3	36.58 ± 1.76	17.30 ± 1.1	13.62 ± 1.01	19.10 ± 1.00	21.34 ± 3.01
4	77.04 ± 2.1	21.13 ± 1.24	19.15 ± 1.0	37.28 ± 2.81	57.29 ± 3.8
5	19.94 ± 1.66	13.84 ± 1.2	7.992 ± 0.81	13.24 ± 1.2	11.46 ± 1.1
6	25.22 ± 1.21	10.99 ± 1.8	10.01 ± 1.4	15.52 ±	13.22 ± 1.3
7	>100	>100	>100	>100	>100
8	>100	>100	>100	>100	>100
9	24.74 ± 2.0	9.40 ± 1.9	9.34 ± 0.56	18.96 ± 1.5	9.78 ± 1.01
10	19.68 ± 1.22	13.71 ±1.1	8.343 ± 0.472	12.1 ± 3.0	13.45 ± 2.09
11	24.89 ± 1.9	8.73 ± 0.21	10.16 ± 1.89	16.30 ± 1.9	13.95 ± 1.3
12	31.76 ± 1.5	9.87 ± 0.71	9.53 ± 1.99	15.76 ± 2.1	11.13 ± 1.8
13	32.57 ± 2.5	12.09 ± 0.99	11.62 ± 0.101	22.79 ± 2.3	21.46 ± 1.7
14	>100	>100	>100	>100	>100
15	>100	43.27 ± 4.01	32.37 ± 1.88	29.55 ± 2.8	50.94 ± 3.51
16	>100	>100	41.44 ± 1.91	67.07 ± 4.1	60.79 ± 4.22
17	>100	>100	99.29 ± 6.16	64.26 ± 5.02	>100
18	>100	>100	>100	>100	>100
19	58.54 ± 3.2	>100	57.28 ± 3.1	69.54 ± 4.23	39.78 ± 3.9
20	78.85 ± 4.4	>100	>100	>100	>100
21	22.61 ± 1.1	11.07 ± 0.4	9.353 ± 1.2	19.84 ± 2.01	17.02 ± 1.7
22	>100	>100	>100	>100	>100
23	16.79 ± 1.76	9.492 ± 0.7	6.873 ± 1.2	12.98 ± 0.54	9.533 ± 1.5
24	14.16 ± 1.0	5.584 ± 0.2	7.149 ± 0.4	11.43 ± 1.0	8.722 ± 1.0
25	14.49 ± 0.2	6.936 ± 0.61	5.251 ± 0.67	7.772 ± 1.1	7.72 ± 1.6

**Figure 2:**
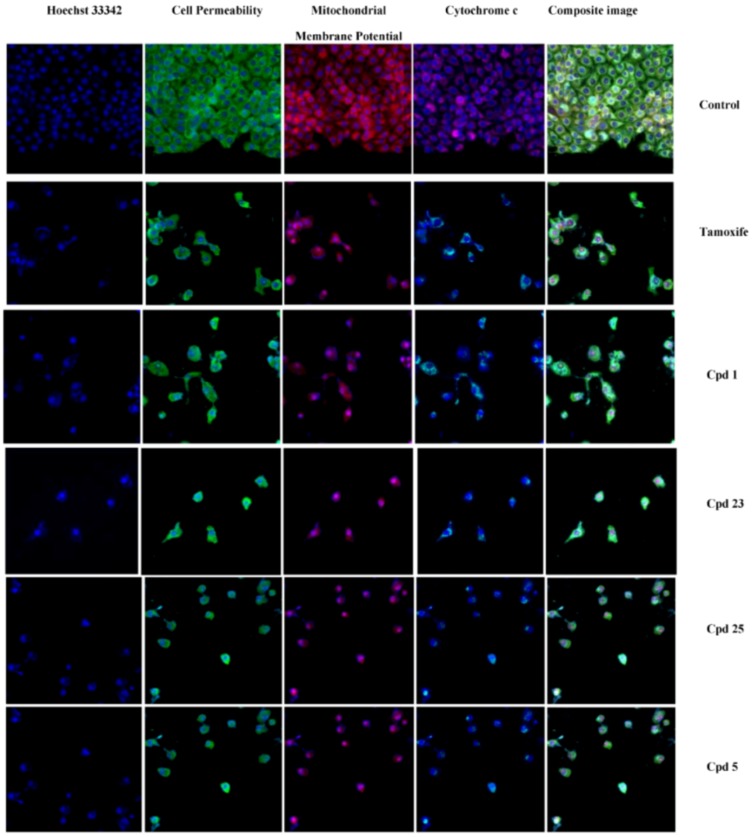
Representative images of MCF7 cells treated with medium alone and compounds, and stained with nuclear staining dye, cell permeability dye, mitochondrial membrane potential dye and cytochrome c. The images from each row are obtained from the same field of the same treatment sample. MCF7 cells produced a marked reduction in mitochondrial membrane potential, and marked increases in membrane permeability and cytochrome c. (Magnification 20X). (1A- 3 µg/mL; 1B- 6 µg/mL), (23A- 3 µg/mL; 23B- 6 µg/mL), (25A- 2 µg/mL; 25B- 4 µg/mL) and (5A- 3 µg/mL; 5B- 6 µg/mL). The positive control used in the analysis was tamoxifen (0.04 µg/mL). “Cpd” refers to compound.

### 2.3. Multiparameter Cytotoxic Analysis

**Figure 3:**
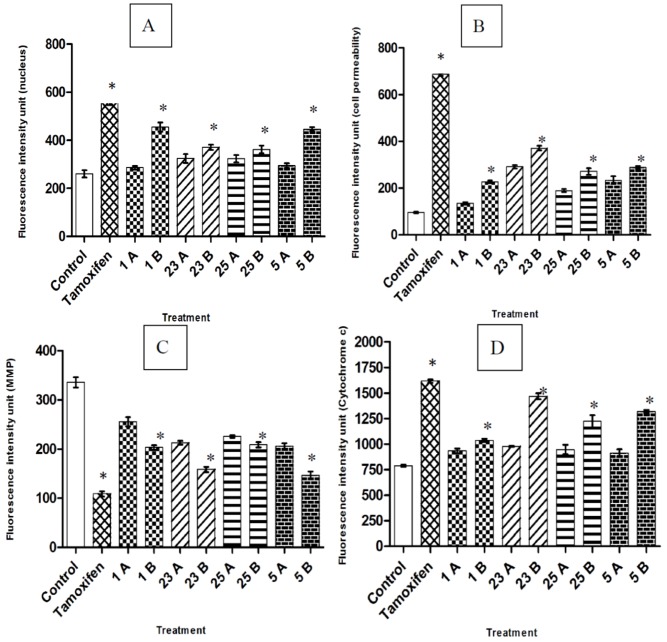
Quantitative analysis of compounds mediated apoptosis parameter. Changes in (**A**) total nuclear intensity; (**B**) cell permeability; (**C**) mitochondrial membrane potential and (**D**) cytochrome c localization were all measured simultaneously in MCF7 cells. Following treatment with compounds 1, 23, 25 and 5, there was statistically significant cell loss (data not shown), increased total nuclear intensity, increased cell permeability, loss of mitochondrial membrane potential and cytochrome c release from mitochondria with good *p* values. (1A- 3 µg/mL; 1B- 6 µg/mL), (23A- 3 µg/mL; 23B- 6 µg/mL), (25A- 2 µg/mL; 25B- 4 µg/mL) and (5A- 3 µg/mL; 5B- 6 µg/mL). The positive control used in the analysis was tamoxifen (0.04 µg/mL). Each experiment was performed at least two times. Results are expressed as the means value ± standard deviation (SD). Statistical analysis was performed with one-way analysis of variance (ANOVA) using GraphPad Prism software (version 4.0; GraphPad Software Inc.). Statistical significance is expressed as *, *p* < 0.05.

Though the chalcones produced growth inhibition, the mode of cell death was yet to be studied. Different modes of cell death include autophagy, apoptosis and necrosis; of which apoptosis is the desired mode of cell death in chemotherapy [21]. As such, studies are extensively focused on the apoptotic potential of a compound to be anticancer agent [5,22]. In the current study, we therefore investigated the involvement of apoptosis in the cytotoxicity produced by select compounds (**1**, **5**, **23** and **25**) in MCF-7 cells by examining the cellular changes associated with apoptosis using HCS analysis. The treated cells exhibited a concentration-dependent increase in nuclear chromatin staining with Hoechst 33342 (Figure 2 and Figure 3) with all compounds, suggesting an increase in nuclear condensation. The increase in nuclear chromatin staining to Hoechst 33342 dye was also observed in tamoxifen positive control cells whereas the fluorescent intensity was significantly lower in the control.

The high fluorescence intensity for the permeability dye in the cytoplasm of chalcone treated and tamoxifen treated cells evidenced the increase in membrane permeability compared to the control cells. A reduction in mitochondrial membrane potential dye retention was observed with increasing concentrations of the compounds used for treatment and with the tamoxifen positive control whereas the control cells had intact mitochondrial membrane potential dye retention. The specific apoptosis indicator, release of cytochrome c was found to increase in concentration dependent manner with all compounds used in the treatment and with the tamoxifen treated cells. Chromatin condensation [23], increased cell permeability [24], decline in mitochondrial membrane potential [25] and cytochrome c release [26] are key events of apoptosis which were obviously observed when the selected compounds were treated on MCF-7 cells. There are previous studies demonstrating that chalcones were good candidates in producing apoptosis in MCF-7 cells [22,27].

### 2.4. Caspase-3/7, -8, and -9 Activity

The caspases, a family of cysteine-dependent aspartate-directed proteases, are prominent among the death proteases [28]. There are two type of caspases, namely upstream (initiator) caspases and downstream (effector) caspases [29]. Upstream caspases, which include caspase-8 and caspase-9 cleave inactive pro-forms of downstream caspases like caspase-3 and caspase-7; which in turn cleave proteins involved in programmed cell death events [30]. In our analysis, following treatment with the selected synthesized compounds, the caspase-3/7, caspase-8, and caspase 9 activity was increased in MCF-7 cells as compared to control. Caspase-3/7 activity of all compounds was more than 100,000 RLU, where the maximum caspase-3/7 activity (210430 RLU) was achieved at highest concentration of compound 1. The control cells, on the other hand, had only caspase-3/7 activity of 30,140 RLU. When the caspase-8 activity of control cells were just 50892 RLU, the highest concentrations of compound 1, 23, 25 and 5 showed significant activity (*p* < 0.05) of 17,568 RLU, 18,342 RLU, 18,356 RLU and 10,435 RLU respectively. Similarly, caspase-9 activity was also elevated in all treatments of the compounds used to analyze. All the compounds except compound **5** showed caspase-9 activity of more than 12,000 RLU. On the whole, all the four compounds showed increase in caspase-3/7, caspase-8, and caspase-9 activity with increase in concentration suggesting that there is the activation of both intrinsic and extrinsic apoptotic pathways [31]. It is also important to note that MCF-7 cells do not have caspase-3 [32], so the activity represented is of caspase-7. Recently, Deeb *et al.*, found that xanthohumol, a chalcone, elicited apoptosis in cancer cells by both intrinsic and extrinsic pathways [33]. Similarly, another study proved that chalcones are capable in inducing both pathways in MCF-7 and MDA-MB-231 breast cancer cell lines [27].

**Figure 4:**
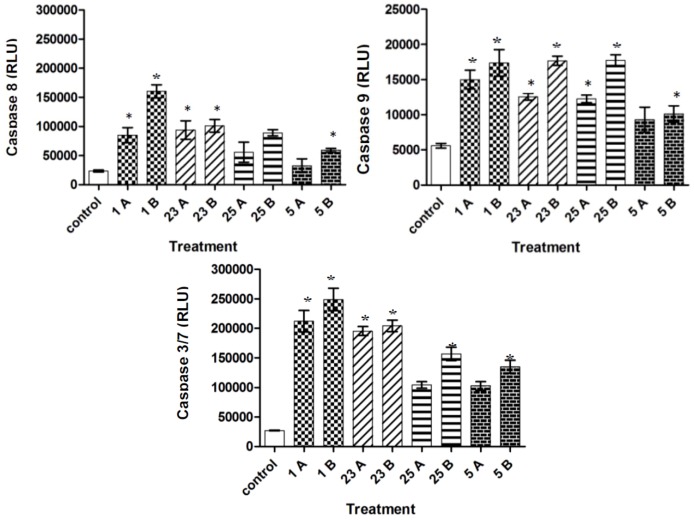
Relative luminescence expression (RLU) of caspase 3/7, 8 and 9 in the MCF7 cells treated with different concentrations compounds. (1A- 3 µg/mL; 1B- 6 µg/mL), (23A- 3 µg/mL; 23B- 6 µg/mL), (25A- 2 µg/mL; 25B- 4 µg/mL) and (5A- 3 µg/mL; 5B- 6 µg/mL). Triplicates of each treatment group were used in each independent experiment. The statistical significance is expressed as *, *p* < 0.05.

### 2.5. Intracellular ROS Level

**Figure 5:**
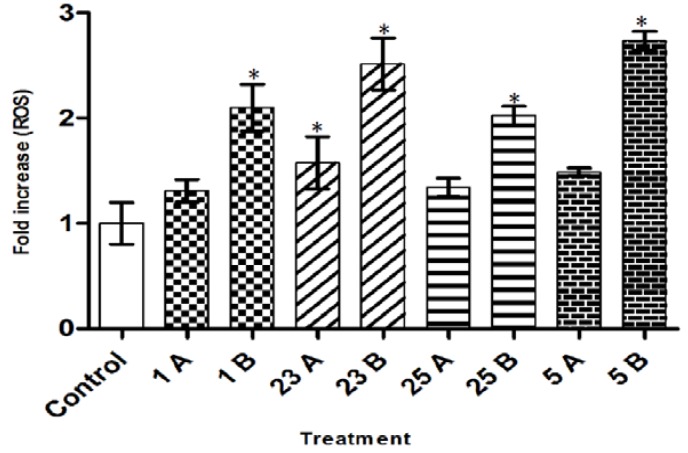
Fold increase of reactive oxygen species (ROS) in MCF-7 cells after treatment with compounds **1**, **23**, **25**, and **5** compared to control (untreated MCF-7 cells). (1A- 3 µg/mL; 1B- 6 µg/mL), (23A- 3 µg/mL; 23B- 6 µg/mL), (25A- 2 µg/mL; 25B- 4 µg/mL) and (5A- 3 µg/mL; 5B- 6 µg/mL). Triplicates of each treatment group were used in each independent experiment. The statistical significance is expressed as * *p* < 0.05.

There was loss of mitochondrial membrane potential (mitochondrial damage) in MCF-7 cells upon treatment with the selected compounds. This could probably be due to the reactive oxygen species (ROS) produced within the mitochondria, which primarily affect the mitochondria itself [34]. The intracellular ROS level was therefore studied with DCFDA and the resulting data showed that all the compounds used in this study significantly increased the production of ROS in MCF-7 cells compared to untreated cells. (Figure 5). All the compounds showed more than 1.3-fold increase (*p* < 0.05) at low concentration and more than 2.0-fold increase (*p* < 0.05) at high concentration as compared to control. Amongst the compounds used, compound **5** showed the highest ROS level (2.7-fold higher) (*p* < 0.05) at the highest concentration used (6 µg/mL). It is thus concluded that ROS production is involved in induction of apoptosis by chalcones, which is consistent with previous findings [35,36,37]. ROS-dependent apoptosis induction by chalcones was observed previously in similar studies where flavokawain B, a novel chalcone, increased the intracellular ROS levels in colon cancer HCT116 cells [35] and oral carcinoma HSC-3 cells [36]. 

## 3. Experimental

### 3.1. Synthesis of Chalcones

Procedure A (compounds **1**, **3**, **4**, **5**, **7–9**, **11–13**, **15–17**, **19–20**): Substituted acetophenone (0.01 mol) and substituted aldehyde (0.01 mol) were mixed in ethanol (40 mL) in a round bottom flask placed in an ice bath. To this NaOH solution (10 mL, 60%) were added dropwise with continuous stirring for 30 min. The mixing was continued for another 2–3 h at room temperature. The mixture was kept in a refrigerator for overnight when it became quite thick. Then it was diluted with ice-cold distilled water (40 mL), filtered, washed well with cold water, dried in air and recrystallized from rectified methanol.

Procedure B (compounds **2**, **6**, **10**, **14**, **18**, **21**, **23–24**): Substituted acetophenone (0.01 mol) and substituted aldehyde (0.01 mol) were mixed in ethanol (40 mL) in a round bottom flask placed in an ice bath. To this NaOH solution (10 mL, 60%) were added dropwise with continuous stirring for 30 minutes. The mixing was continued for another 2–3 h at room temperature. Turbidity appeared in the mixture, which was then diluted with cold distilled water (40 mL), and neutralized to litmus paper with 2 N HCL. The product (ppt) was filtered, washed well with cold water, dried in air and recrystallized from rectified methanol.

Procedure, C (compound **22**): Substituted acetophenone (0.01 mol) and substituted aldehyde (0.01 mol) were mixed in ethanol (40 mL) in a round bottom flask placed in an ice bath. To this NaOH solution (10 mL, 60%) were added dropwise with continuous stirring for 30 min. The mixture was refluxed overnight. The stirring of the mixture was continued for 2 days at room temperature (turbidity appears in the mixture), then the mixture was diluted with cold distilled water (40 mL), and neutralized to litmus paper with 2 N HCL. The product (ppt) was filtered, washed well with cold water, dried in air and recrystallized from rectified methanol.

### 3.2. Identification of Chalcones

The IR spectra were obtained using a Shimadzu IR-FTIR-8300 spectrophotometer in a disk of potassium bromide, over the range of 4,000–400 cm^−1^. The ^1^H-NMR spectra were recorded on a Varian Mercury-VX-200 (300 MHz) spectrophotometer using DMSO-d_6_ as solvent and TMS as the internal standard. The melting temperatures were determined in sealed capillaries on a SMP3 melting point apparatus (Stuart Scientific, UK).

### 3.3. Cell Culture and Cell Viability Assay

A549, PC3, MCF-7, HT-29, WRL68 and MCF-7 cells were obtained from the ATTC (Rockville, MD, USA) and grown in RPMI-1640 medium, 10% fetal bovine serum (FBS) and 1% pen-strep, in humidified conditions at 5% CO2 and 37 °C. The different cell lines mentioned above were used to determine the inhibitory effects of compounds on cell growth using the MTT assay. This colorimetric assay is based on the conversion of the yellow tetrazolium bromide (MTT) to a purple formazan derivative by mitochondrial succinate dehydrogenase in viable cells. Cells were seeded in 96-well plate and incubated for 24 h at 37 °C with 5% CO_2_ saturation. After incubation, the cells were treated with different concentration of compounds and incubated for another 24 h. After 24 h of drug treatment, 20 µL of MTT solution at 5 mg/mL was added and incubated for 4 h. Dimethyl sulfoxide (DMSO) in volume of 100 µL is added into each well to dissolve the purple formazan formed. The colorimetric assay is measured and recorded at absorbance of 570 nm. 

### 3.4. Cell Treatment

Due to the observation of significant cytotoxicity of selected compounds towards MCF-7 cells, the following analyses were done on them only. Cells were treated with compound 1 (1A- 3 µg/mL; 1B- 6 µg/mL), 23 (23A- 3 µg/mL; 23B- 6 µg/mL), 25 (25A- 2 µg/mL; 25B- 4 µg/mL) and 5 (5A- 3 µg/mL; 5B- 6 µg/mL).

### 3.5. Multiparametric High Content Screening (HCS) Assays

The toxic effects of Cpd **1**, **5**, **23** and **25** at different doses on MCF-7 cells were assayed using the high content screening (HCS) KineticScan Reader (Cellomics Inc, Pittsburgh, PA, USA) with the Multiparameter Cytotoxicity Kit 3. The kit contains four fluorescent dyes, *i.e.*, blue fluorescent Hoechst 33342, membrane permeability, mitochondrial membrane potential, and cytochrome c dyes. They respectively detect changes in nuclear morphology (nuclear condensation), membrane permeability, mitochondrial membrane potential and expression of cytochrome c. The multiparametric apoptosis kit quantifies three fundamental parameters related to the process of apoptosis, *i.e.*, nuclear condensation detected by the blue fluorescent nuclear dye, Hoechst 33342, cell permeability detected by permeability dye, mitochondrial damage detected by mitochondrial membrane potential dye and cytochrome c release by DyLight 649 Conjugates. Following treatment with different concentrations of compounds 1, 23, 25 and 5 as mentioned before, the fixation and staining for imaging analysis of the MCF-7 cells were performed according to the manufacturer’s instructions. Cells treated with tamoxifen (0.04 µg/mL**)** were used as positive control and untreated cells were used as negative control. Plates were analyzed using a Thermo Scientific ArrayScanHVTI HCS Reader (Cellomics Inc, Pittsburgh, PA, USA). This is a computerized automated fluorescence imaging microscope that automatically identifies stained cells and measures the intensity and distribution of fluorescence in individual cells. Images for each fluoroprobe were acquired at different channels using suitable filters with 20x objective at fixed exposure time. The Cell Health Profiling BioApplication software was used for image acquisitions and analysis. For each well, at least 25 fields, corresponding to at least 500 cells were automatically acquired and analyzed. All experiments were performed in triplicates. Cell average intensity (Mean) under the modified object mask within selected range in each channel was used as assay indicator, and reported as average fluorescence intensity.

### 3.6. Caspase Activity

Activity of cellular caspases was measured using the Caspase-Glo-9, Caspase-Glo-3/7 and Caspase-Glo-8 assay reagents (Promega), according to the manufacturer's instructions. The assays provide a luminogenic caspase-3/caspase-7 or caspase-8 or caspase-9 substrate, respectively, in a buffer system optimized for caspase activity measurement in cell lysates. The luciferase signal generated is proportional to the amount of caspase activity present. MCF-7 cells have no caspase-3 activity due to functional deletion of the CASP-3 gene [38]. Therefore, the activity measured in this test is that of caspase-7. Briefly, cells were plated in opaque 96-well plates in triplicate and treated with the. After 24 h treatment with compounds **1**, **23**, **25** and **5** as mentioned before, the assay reagents were added to the plates at room temperature and incubated for 60 min, and the luminescence was measured using a plate reader. All assays were done in triplicate, and means ± SD were calculated for each condition.

### 3.7. Intracellular ROS Level

The production of ROS was estimated by fluorescence using the redox-sensitive fluorescent probe DCFDA (Sigma). The oxidation of DCFDA by ROS converts the molecule to 2′,7′-dichlorodihydrofluorescein (DCF), which is highly fluorescent. Thus, ROS production by stimulated cells causes an increase in the fluorescence signal over time. ROS assays were performed as previously described (13). Briefly, the MCF-7 cells were seeded in 96-well plates (5 × 10^4^ cells/well) and allowed to attach for 24 h. After 24 h treatment with compounds **1**, **23**, **25** and **5** as mentioned before, the cells were washed three times with PBS and incubated for 60 min with five DCFDA at room temperature. Again the wells were washed with PBS. Fresh PBS was added (100 μL/well) and DCF fluorescence was detected using a Tecan Infinite M200 fluorescence microplate reader at excitation and emission wavelengths of 485 and 528 nm, respectively. The fluorescence intensity unit of treatments was compared with control.

### 3.8. Statistical Analysis

All assays were conducted in at least three separate experiments. Results are expressed as the mean value ± standard deviation (SD). Statistical analysis was performed with one-way analysis of variance (ANOVA) using GraphPad Prism software (version 4.0; GraphPad Software Inc., San Diego, CA, USA). 

## 4. Conclusions

In summary, we have synthesized some chalcones and identified them from their spectral data. We have nhat most of the compounds were very active cytotoxic agents towards several cancer cell lines. Four active compounds chosen in the study showed apoptosis induction in MCF-7 cells with the involvement of caspase-7, caspase-8, and caspase-9. We also demonstrated that all four compounds used in the study induced ROS generation and loss of mitochondrial membrane potential to cause the release of cytochrome c to result in apoptosis. The apoptosis was through both intrinsic and extrinsic pathways. 
